# Gut Microbiota Implications for Health and Welfare in Farm Animals: A Review

**DOI:** 10.3390/ani12010093

**Published:** 2021-12-31

**Authors:** Siyu Chen, Shuyan Luo, Chao Yan

**Affiliations:** 1 Guangdong Provincial Key Laboratory of Animal Molecular Design and Precise Breeding, School of Life Science and Engineering, Foshan University, Foshan 528225, China; chensiyu@fosu.edu.cn (S.C.); shuyenlo@163.com (S.L.); 2 Shenzhen Branch, Guangdong Laboratory for Lingnan Modern Agriculture, Genome Analysis Laboratory of the Ministry of Agriculture, Agricultural Genomics Institute at Shenzhen, Chinese Academy of Agricultural Sciences, Shenzhen 518124, China

**Keywords:** farm animal, fecal microbiota transplantation, gut microbiota, welfare

## Abstract

**Simple Summary:**

Farm animal health and welfare have been paid increasing concern in the world, which is generally assessed by the measurements of physical health, immune response, behavior, and physiological indicators, such as stress-related hormone, cortisone, and norepinephrine. Gut microbiota as a “forgotten organ” has been reported for its great influence on the host phenotypes through the immune, neural, and endocrine pathways to affect the host health and behavior. In addition, fecal microbiota transplantation as a novel approach is applied to regulating the composition and function of the recipient farm animals. In this review, we summarized recent studies that gut microbiota influenced health, immunity, behavior, and stress response, as well as the progress of fecal microbiota transplantation in farm animals. The review will provide new insights into the measurement of farm animal health and welfare concerning gut microbiota, and the implication of fecal microbiota transplantation to improve productivity, health, and welfare. Above all, this review suggests that gut microbiota is a promising field to evaluate and improve animal welfare.

**Abstract:**

In the past few decades, farm animal health and welfare have been paid increasing concern worldwide. Farm animal health and welfare are generally assessed by the measurements of physical health, immune response, behavior, and physiological indicators. The gut microbiota has been reported to have a great influence on host phenotypes, possibly via the immune processes, neural functions, and endocrine pathways, thereby influencing host phenotypes. However, there are few reviews regarding farm animals’ health and welfare status concerning the gut microbiota. In this point of view, (1) we reviewed recent studies showing that gut microbiota (higher alpha diversity, beneficial composition, and positive functions) effectively influenced health characteristics, immunity, behaviors, and stress response in farm animals (such as pigs, chickens, and cows), which would provide a novel approach to measure and evaluate the health status and welfare of farm animals. In addition, fecal microbiota transplantation (FMT) as one of the methods can modulate the recipient individual’s gut microbiota to realize the expected phenotype. Further, (2) we highlighted the application of FMT on the improvement of the production performance, the reduction in disease and abnormal behavior, as well as the attenuation of stress in farm animals. It is concluded that the gut microbiota can be scientifically used to assess and improve the welfare of farm animals. Moreover, FMT may be a helpful strategy to reduce abnormal behavior and improve stress adaption, as well as the treatment of disease for farm animals. This review suggests that gut microbiota is a promising field to evaluate and improve animal welfare.

## 1. Introduction

What is animal welfare? According to Fraser et al. (1997) [1], the understanding of animal welfare was mainly three types: (1) that animals should lead natural lives through the development and use of their natural adaptations and capabilities; (2) that animals should feel well by being free from prolonged and intense fear, pain, and other negative states, and by experiencing normal pleasures; (3) that animals should function well, in the sense of satisfactory health, growth, and normal functioning of physiological and behavioral systems. As far as animal welfare is concerned, objectively and scientifically assessing animal welfare has become an essential issue for an animal producer, stock person, and consumers. Many terminologies and measurements are well established in farm animal welfare and behavioral science. To date, health conditions, immune response, behavior, and physiological indicators related to stress are readily and widely used in this area [2,3,4,5]. Recently, mathematics, including multi-linear models, regression analysis, polynomial model, and calculus, is considered an ideal tool for precise detection and measurement of an animal’s response to good and bad welfare [6].

In recent years, gut microbiota, having a profound impact on the host’s health, is a great concern and referred to as a forgotten organ [7]. There is a large number of microorganisms inhabiting the intestinal tract of humans and animals [8], with more than 10 times the number of human and animal cells and 150 times as many genes as the host genome [9,10]. The phylogenetic diversity of gut microbiota is harbored in production animals, such as the estimation of 375 phylotypes (a phylogeny having taxa or strains annotated with extrinsic traits) in pigs [11], the range from 300 to 1000 bacterial species in the cow rumen [12], approximately 915 operational taxonomic units (the assigned 97% sequence similarity by 16S rDNA bioinformatic analysis) in chickens [13,14], and the range from 2000 to 3000 operational taxonomic units in sheep [15]. The gut microbiota is distinct in different intestinal tracts, such as in jejunum, ileum, and cecum, regions (the mucosal microbiota to luminal microbiota), and growing periods (from early life to adult) in density and diversity [16]. A vast and diverse microbial ecosystem is essential for the health of humans [17], dairy [18], and chickens [19]. The gut microbiota is affected by many factors, including age [20], diet [21], rearing system [22], and so on. Further, the role of gut microbiota is being studied in neuroscience and a concept of the microbiota-gut-brain axis has emerged and is being explored [23]. The microbiota-gut-brain axis, namely a bidirectional communication among neural, hormonal, and immunological routes [23], is linked to gut inflammation [24] and alternations of stress and behavioral responses [25,26]. This bidirectional communication, to some extent, could address the activities of brain function (a stress-related hormone) with the immune response through the activities of gut microbiota and, consequently, behavioral response.

**Figure 1:**
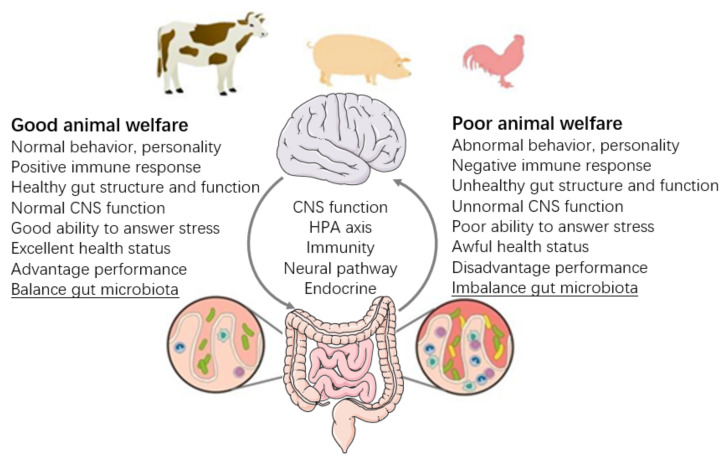
Animal welfare in the implication and perspective of the gut microbiome.

A recent study has reviewed the gut microbiota and its impact on brain development and behaviors in laboratory and human studies, which provides a better understanding and functional implications of this relevance on farm animals [27]. In this review, we will provide direct evidence of interactions of gut microbiotas as a welfare indicator and the application of fecal microbiota transplantation (FMT) on farm animals. Particularly, (1) we reviewed recent studies showing that gut microbiota influenced health, immune response, behavior, and stress in farm animals, which would provide a novel approach to measure the welfare of farm animals in terms of the gut microbiota (diversity, composition, and functions). Further, (2) we highlighted the application of FMT on the decrease in disease and abnormal behavior and the attenuation of stress in farm animals. In particular, we would provide new insight into the microbiota-gut-brain axis (Figure 1 referred to [23]) to systemically evaluate animal welfare. 

## 2. Gut Microbiota and Health of the Hosts

Previous studies have demonstrated that the gut microbiota plays a crucial role in the absorption and metabolism of feed [28,29], regulating gut motility and intestinal barrier homeostasis [30,31,32]. To elicit a well-functioning and healthy gut, the dynamic balance of the gut ecosystem is of importance.

A balance between beneficial and harmful bacteria in the gut (at least 85% of total bacteria should be beneficial bacteria, which is highly specific for health and production performance) is vital for the host [33]. The commensal bacteria contribute to the health of chickens [34], pigs [35], cows [18], sheep [36], horses [37], quails [38], and so on. This is because of the role of the bacterial metabolic end-products of short-chain fatty acids from the fermentation of dietary fiber, resistant starch, principally acetate, propionate, and butyrate [39]. These end-products play an indispensable role in regulating energy homeostasis and other physiological purposes [40] and influence the activity of digestive enzymes. Moreover, microbial metabolites can communicate with the gastrointestinal mucus system to influence intestinal homeostasis and neurological disorders [41]. On the contrary, diseases are usually accompanied by an imbalance of gut microbiota composition.

Gut microbiota is closely associated with host health. For example, a study compared Tibetan chickens, a typical breed habituating in high-altitude regions which are speculated to have a unique gastrointestinal microbiota, with a commercial breed Lohmann egg-laying hen and a local breed Daheng broiler chicken on their gut microbiome and diseases [42]. The results indicated Tibetan chickens had a specific abundance of microbes with less pathogen-related microbes, while layers and broiler chickens indicated higher mucosal inflammation risks in the intestinal tracts. In our previous study, we reared dual-purpose chickens for meat and laying with three different diets, namely basal feed, routine feed with 0.6% partial replacement of soybean with dried mealworms, and basal feed with partial replacement of dried mealworms and additional fresh grass [43]. Chickens that had mealworms in their diet showed increased alpha diversity compared with those fed only routine feed, suggesting a potential healthier status of mealworm-fed chickens. Besides, microbiota composition changes are evident in slow- or fast-growing chickens [44], which may be related to different biology in chickens. In pigs, gut microbiota acts as a leading cause in the process of post-weaning diarrhea and associated infections [35] and improves health and production [45]. Similarly, the gut microbiota is associated with weaning in horses [37]. Diarrheic calves are associated with dysbiosis and changes in the predictive metagenomic function of the bacterial communities [46]. Besides, gut microbiota accounts for the emotional reactivity in the Japanese quails [38]. Even though the mechanism and interaction between physiological characteristics and gut microbiota of the host are not yet known, the gut microbiota, to some extent, serves as an indicator of animal health, which is deemed to be linked with productivity [27]. In addition, as Kraimi’s review indicates, gut microbiota is a key factor for evaluating welfare, including: in the relationships with anxiety-like behavior in humans, rodents, turkeys, quails, and horses; on memory capacities in humans, rodents, quails, and pigs; on social behavior in humans, rodents, chickens, turkeys, and quails; on feeding behavior in rodents, goats, cows, chickens, and turkeys [27].

Thus, this insight has the potential to contribute much to our understanding and assessment of the gut microbiota, health, and different welfare states in farm animals. Even farm animals such as chickens, whose gut microbiota breed-specific variations, in terms of operational taxonomic units and composition, suggest scope for quantitative genetic analysis and the potential for selective breeding in chickens for defined gut microbiota [47].

## 3. Gut Microbiota and Immune Indicator of Hosts

The gut immune system, containing 70–80% of the whole body’s immune cells [48], plays a profound impact on the development of hosts, including the development of innate and adaptive immune responses [49,50]. Accumulating data proved that gut microbiota regulated and fine-tuned the immune system throughout life [51,52]. Multiple innate immune cell subsets have been identified in both murine and human intestinal lamina propria. The authors demonstrated that commensal bacteria are capable of directly affecting innate and adaptive immune systems. Importantly, the resident microbiota is recognized to suppress unnecessary inflammatory responses, thereby helping to maintain immune homeostasis [53].

As known, germ-free (GF) mice have been widely used to study the gut microbiota and immune system [54]. In farm animals, the gut microbiota also plays a critical role in the development of the intestinal immune system, while, in turn, the immune system shapes the gut microbiota in chickens [51,52,53,54,55]. Early studies with GF chickens have demonstrated that gut microbiota is essential for the immune system, even though the weights of immune organs (bursa, thymus, or spleen) showed no consistent differences between GF birds and conventional birds [56]. Similarly, a study compared GF chickens, Ross 308 broilers, with conventional birds and revealed that the absence of gut microbiota affected neutral and acidic goblet cell number and density, sialylated and sulfated acidic mucin staining, and MUC2 expression at 7 d of age, indicating a less developed intestinal mucosa in GF birds [57]. A previous study indicates that feeding prebiotic galacto-oligosaccharides can increase cytokine immune effectors interleukin-17A (IL-17A) gene expression counterposed to a decrease in IL-10 concerning the innate immune responses in broilers [58]. Moreover, compared to strict hygienic conditions, chicks exposed to maternal feces after hatching can increase the levels of IgA and IgY to influence the immune responses in chickens [59]. In pigs, the administration of *Lactobacillus rhamnosus GG* in piglets can promote the early B lineage development, influence the Ig CDR3 repertoires composition of B cells, and promote the IgA production in the gut lamina propria [60]. Besides, the antibody response is delayed in GF piglets due to immature immune structures [61]. The strain of commensal *Escherichia coli* had a significant effect on the immune structure and resulted in the extensive recruitment of T cells to epithelium and lamina propria, compared with GF piglets [62]. Most notably, GF animals are widely used to study phenotype modifications, but it is not easy to demonstrate that phenotype changes are attributed to the absence of gut microbiota rather than physiological alterations. Besides, a study revealed that different feed consumptions change calf bacterial diversity and expression of genes encoding host mucosal immune responses in dairy calves during weaning transition [63]. Furthermore, gut immune maturation relies on a coevolved host-specific microbiota in GF mice [64].

## 4. Gut Microbiota and Behavior of Hosts

There is a communication between the gut microbiota and the central nervous system, although the mechanisms of gut microbiota mediating the microbiota-gut-brain axis to influence behavior are not fully understood [23,65]. Despite bodies of studies on the understanding of factors affecting gut microbiota, large gaps remain in the contribution of microbial ecology to animal behavior. Gut microbiota, serving as a critical role in modulating social and affective behaviors, including aggression, investigation, and depressive- and anxiety-like behaviors, is mainly conducted on laboratory animals and non-human primate animals [66,67,68].

Given rare studies of farm animals on this relevance, we first summarized work done in social animals, including non-human primate animals, vertebrates, and birds, which generated an implication on farm animals. A critical view has been emerging that microbiota shapes the host phenotype. Bacteria are common infectious agents, and most bacteria are transmitted through close contacts or by intermediates like foods, water, air, and objects in the environment between individuals, especially in mammals [69,70]. In Japanese quails, the absence of gut microbiota reduces emotional reactivity relating to fear and social perturbation [38]. The social organization and behavioral patterns were also known to transmit bacterial communities [69,71]. Besides, social relationships could shape bacterial transmission in vertebrates and birds. For instance, in four-toed salamanders, eggs in communal nests were more likely to have beneficial, antifungal bacteria than those in solitary nests, which, in turn, contributed to higher embryonic survival and lower catastrophic nest failure [72]. In bluebirds, plumage bacteria intensity in nesting pairs was significantly positively correlated, suggesting that birds sharing the same nest transmit bacteria to each other [73], and birds infected with *Salmonella* lead to less active feeding and drinking activities [74]. Again, social contact among social animals is likely to influence the microbiota of the host, which, in turn, affects host phenotypes. More recently, GF quails showed reduced fearfulness to those colonized with gut microbiota [38]. In chickens, high and low levels of feather pecking in laying hens were associated with intestinal microbial metabolites [75] and the gut microbial community. That is, high levels of feather pecking birds were characterized by a higher diversity and evenness of microbiota, as well as a relative abundance of genera of *Clostridiales* (belonging to the order of *Clostridia*), but a lower relative abundance of *Staphylococcus* spp. and *Lactobacillus* spp. compared to low levels feather pecking birds. However, further investigations are needed to discover the causality and mechanism of the relationship of feather pecking with gut microbiota [76,77]. Furthermore, we compared free-range and cage-reared hens, of which those free-ranged hens were living with roosters with social contact with other mates, while those caged hens were reared in a single cage with limited contact with the other hens. We found that the hens living with roosters showed an upregulated gonadotropin-releasing hormone pathway in the gut microbiome as compared with the control subjects, which may implicate that social contact is associated with the gut microbial functions [22]. Besides, feather bacterial load in pigeons had been proved to adjust preening [78]. In pigs, gut microbiota has a profound effect on the porcine appetite and feeding behavior [79]. A maternal western diet during gestation and lactation, even in the absence of obesity, has significant consequences for piglets’ blood lipid levels, microbiota activity, microbiota–gut–brain axis, and neurocognitive abilities after weaning [80,81]. Moreover, in beef cattle, the maternal grooming behavior reduces the bacteria in calf coats [82]. Thus, it is reasonable that free-range chickens showed more similarities of beta-diversity of gut microbiota within individuals than those reared under the cage [22,43].

According to the above literature, social contacts of social animals contribute to the alternation of gut microbiota; nevertheless, the underpinned mechanism needs to be explored. Moreover, it appears there is a mix between gut microbiota influences on behavior and the influences of behavior on gut microbiota. Accumulating experimental approaches evaluate the behavior as a consequence of manipulating the microbiome through direct (e.g., vagus nerve) and indirect (e.g., hormones, cytokines, and fatty acids) mechanisms. The field of microbiology expands our understanding of the interface of complex gut microbiota and animal behavior. However, to what extent, and how, is animal behavior driven by the microbiome (the genes, genomes, and products of the microbiota, as well as the host environment [83])? The question is needing more study to answer. 

## 5. Gut Microbiota and Stress of Hosts

Gut microbiota plays a vital role in the process of stress response [84]. Nurturing an optimal gut microbiome may indicate positive and beneficial effects in animal science as a means to manage stressful situations and to increase the productivity of farm animals [85]. It has long been known that stress and the associated activity of the hypothalamus-pituitary-adrenal (HPA) axis can influence the gut microbial composition [86,87]. The HPA axis modulates cortisol and corticosterone secretion, while cortisol or corticosterone systemically regulate the immune circulating cytokine secretion in the gut, which can further influence intestinal barrier function and alter gut microbiota composition through the microbiota–gut–brain axis [23,65].

Advances in the understanding of gut microbiota and the HPA axis are mainly conducted on laboratory animals [88,89]. A landmark research conducted in mice suggested that gut microbiota has altered the HPA axis function under stress, accompanied by increased plasma adrenocorticotropic hormone and corticosterone levels, which was reversed by reconstitution with *Bifidobacterium infantis* [87]. Maternal separation, as an early life stressor, was demonstrated to involve HPA axis activity in many species [90,91,92] and was further known to result in a substantial decrease in fecal *Lactobacilli* 3 d after the initiation of the separation procedure in rhesus monkeys [92]. Altered fecal microbiota composition was found in adult rats that had undergone maternal separation for 3 h per day from postnatal days when compared with the non-separated individuals [52]. A study using deep-sequencing methods demonstrated that the composition of microbiota from mice exposed to chronic restraint stress (a physical stressor) differed from that in non-stressed control mice [24]. Specifically, exposure to chronic psychosocial stress decreased and increased the relative abundance of *Bacteroides* spp. and *Clostridium* spp., respectively, in the rat caecum [24]. Similarly, exposure to physiological stress is seen to change the abundance of family *Anaerolineaceae*, genus *Clostridium*, and genus *Oscillibacter* of gut microbiota in western lowland gorillas [93]. The gut microbiota is also related to the brain plasticity of the host in response to the stress in mice [25,87]. In the dairy cow, exposure to heat stress influenced the HPA activity, which is associated with the changes of plasma cortisol, oxytocin concentration, and circulation of cytokines, as well as decreased alpha diversity in gut microbiota [18]. Similarly, in chickens, heat stress led to the alternation of gut microbiota composition and alpha diversity [94]. Our previous study suggested that the stress-inducing cage rearing has decreased the alpha diversity in gut microbiota and downregulated immune-related pathways while upregulating pathogen-related pathways [22]. Particularly, our previous study indicates that perches and litter materials enriched environments, improving gut microbial functions possibility through the HPA axis [95]. Besides, gut microbiota mediated by antibiotics or prebiotics is controlling stress-induced hypertension through modifying the HPA axis in the rats [96]. In addition, stress also can lead to a change in gut homeostasis and a weakened immune system, which increases the risk for colonization by pathogenic bacteria in broiler production [97].

Accumulating evidence indicates that gut microbiota is a cross-talk with the brain through the neural, immune, and endocrine to regulate brain function and behavior. Accordingly, despite the evidence being rare, especially for farm animals, gut microbiota is also a cross-talk connecting the HPA (stress), immune response, and behaviors.

## 6. Application of FMT

FMT, also called stool/fecal transplantation or fecal bacteriotherapy, transplants the fecal material from one individual to another for a desired physiologic effect to manage the reconstruction of gut microbial composition and function in human and non-human beings. Feces also harbors additional substances (proteins, bile acids, and vitamins) which might contribute to the recovery of gut function. Indeed, FMT suggests that feces contain a superior combination of intestinal bacterial strains and are more favorable for the management of reconstruction of gut microbiota by introducing a complete, stable community of intestinal microorganisms. The microbial function is to protect the intestinal tract by directly competing with the host for limited nutrients, regulating host immune response, increasing the resistance to pathogens and potentially harmful bacteria colonization in the intestine, and reconstructing the homeostasis of the intestine [98,99]. The FMT normalizes the composition and functionality of gut microbiota [100,101,102] and has now become widely adopted into clinical treatments for diseases. However, the underpinned mechanism of the FMT on the disease treatment is not yet clear. The potential of the FMT mechanism might be the repair, replacement, and reconstruction of the primary microbiota of hosts by the healthy fecal microbiota [103].

FMT is not a state-of-the-art method. A similar procedure was first applied approximately 1,700 years ago by a Chinese medical scientist named Ge Hong, recorded in an ancient book, Ben Cao Gang Mu, describing Chinese medicine [104]. At that time, patients who had food poisoning or severe diarrhea were treated in terms of oral administration of human fecal suspension. Other important events using FMT in history are well described in a previous review [105]. The FMT was listed as clinical guidelines and has been recommended for the treatment of recurrent *Clostridium difficile* (*Clostridioides difficile*) infection in the US since 2013 [106], indicating landmark progress of the method in the medical field. FMT was also applied on multiple organ dysfunction syndromes, a disease targeting the organ of the gut. As compared with healthy people, the relative abundance of *Firmicute* and *Bacteroides* greatly decreased in patients, while that of conditionally pathogenic bacteria, Proteobacteria, increased. The change of microbial composition is not clearly responsible for the mediator or marker of this disease, which did provide a new strategy for the treatment of patients with gut disease by changing the flora ecology and diversity [107]. However, the application of FMT in the treatment of ulcerative colitis is not consistent, which is probably due to the different methods between oral administration and nostril tube treatment [108,109]. Increasing evidence has suggested that the dysfunction of gut microbiota is associated with autism [110,111]. The clinical application of FMT was further used on more human diseases, including HIV therapy and psychological-related diseases [112,113].

### Application on Behaviors of Laboratory Animals

The FMT normalizes the composition and functionality of gut microbiota [100,101,102] and has now become widely adopted into clinical treatments for diseases. For instance, GF mice displayed more depression-like behaviors after FMT with ‘depression microbiota’ derived from major depressive disorder patients compared with those transplanted with ‘healthy microbiota’ derived from healthy control individuals [114]. Similarly, pieces of evidence showed that the transfer of lean mice feces to obese mice altered obese mice bacteria species diversity and richness [115]. GF mice implanted with the fecal microbiota from irritable bowel syndrome with diarrhea showed faster gastrointestinal transit, intestinal barrier dysfunction, innate immune activation, and anxiety-like behavior compared with those transplanting feces from healthy individuals [116]. A GF pig is an ideal model for human disease due to the low reliability of disease using the mice model and the ethical concerns of the non-human primates. Besides, the FMT of GF pigs was widely used in gastrointestinal pathology and neurology fields [117,118].

Recently, FMT has been applied to farm animals. The FMT could change recipients’ fermentation parameters and bacterial profiles [119] and withdraw the antibiotic-disturbed gastrointestinal microbiota of cattle [120]. Besides, bovine mastitis with dysbiosisof intestinal microbiota was transplanted with fecal microbiota into GF mice inducing the corresponding phenotype. The results showed that mastitis symptoms in the mammary gland, as well as inflammations in a wide range of tissues, including serum, spleen, and colon, were found in the mice [121]. This study provides novel insights into the disease–health–production application. That is, using the fecal microbiota of healthy individuals in those “unhealthy” individuals is considered a perceptive treatment in farm animals. For example, FMT from healthy Congjiang miniature piglets (a Chinese native pig breed known to have a stronger resistance to early weaning, stress-induced diarrhea ability) to the recipients, a commercial breed, significantly prevented early weaning stress-induced diarrhea regardless of the dose [122]. In pigs, FMT plays a critical role in enhancing metabolism [123,124], regulating intestinal mucosal function and alleviating barrier injury [125], and influencing growth performance [126]. Similarly, the administration of the fecal microbiota from healthy chickens has been used to transfer colonization resistance against *Salmonella* to newly hatched chickens [127]. Inoculating the surfaces of incubating eggs with cecal contents from highly or poorly feed-efficient donor chickens has been shown to reduce bird-to-bird variation in microbiota composition [128]. Furthermore, the administration of the FMT from highly feed-efficient donors during the early stages of life could improve feed efficiency [129]. In our recent study, transferring fecal microbiota from broilers with positive physiological functions and behaviors to chicks can improve fearfulness, intestinal morphology, and microbial composition [130]. Moreover, transferring gut microbiota can influence emotional reactivity in Japanese quails [131]. The FMT has been made in the amplification effect of farm animals, and this has had and continues to have an immense impact on our understanding of host–microorganism interactions (summarized as Table 1). More importantly, the FMT is considered to reduce disease, abnormal behaviors, and stress, such as mastitis, feather pecking, wean stress, etc.

The donor of fecal microbiota for the treatment of human disease is extremely strict, considering kinship of donor and received individuals, the drug and disease history, and infectious pathogen examination of the donor [132]. This contributes to the high cost of FMT treatments, which is also one of the limiting factors on the application of farm animals. Besides, as mentioned above, the different administration of fecal microbiota results in different therapy of ulcerative colitis. Up to date, there is no generally accepted best method for transplantation approaches. For the treatment of humans, gastrointestinal routes, including endoscopy, nasogastric tube/nasointestinal tube, and oral pill, or a combination of the above, are mainly used [133,134,135]. For farm animals, the administration of FMT is through oral delivery, feeding, drinking, or stomach tubes. The measurements of FMT in farm animals are rough compared to humans, but it is a promising method to improve gut microbiota in farm animals.

**Table 1:** Fecal microbiota transplanting applied in farm animals.

Species	Context of Study	Delivery Ways	References
Pig	Convey gut characteristics (microbiota composition, intestinal morphology, and physical index) from pigs to mice.	Intragastric gavage	[136]
	Transfer obese pig fecal microbiota to GF mice induces similar characteristics on skeletal muscle development and lipid metabolic profiles.	Nasogastric tube	[124]
	Transfer adult pigs’ fecal microbiota to crossbred newborn piglets to influence piglets’ growth performance, intestinal barrier function, and immune system.	Oral inoculation	[126]
	Transfer healthy pig fecal microbiota to piglets to prevent early weaning, stress-induced diarrhea.	Oral gavage	[122]
	Transfer different breed healthy piglets’ fecal microbiota to newborn piglets to enhance tryptophan metabolism and reduce epithelial injury susceptibility.	Oral inoculation	[123]
	Transfer fecal microbiota of healthy adult pigs to newborn piglets to regulate intestinal mucosal function and alleviate barrier injury.	Oral inoculation	[125]
Chicken	Transfer fecal microbiota of normal adult cocks to newly-hatched chicks to administrate colonization resistance against *Salmonella*.	Ingesta	[127]
	Transfer highly or poorly feed-efficient chicken fecal microbiota to baby chicks to explore the feed efficiency of chicks.	Drinking	[129]
	Transfer fecal microbiota from with positive physiological functions and behaviors of broilers to improve behavior, intestinal morphology, and gut microbiota.	Oral inoculation	[130]
Cow	Transfer rumen content to recipient cow to explore recipients’ fermentation parameters and bacterial profiles.	Feeding	[119]
	Cow to mouse fecal transplantation suggested intestinal microbiome as one cause of mastitis.	Oral administration	[121]
	Transfer cow fecal microbiota to withdrawal antibiotic-disturbed gastrointestinal microbiota.	Rumen fistula	[120]
Steer	Transfer highest or lowest residual feed intake rumen digesta exchange to steer to improve feed efficiency.	Rumen cannulation	[137]
Ruminants	Rumen transfiguration to treat indigestion	Stomach tube	[138]

## 7. Conclusions and Perspectives

Gut microbiota (higher alpha diversity, beneficial composition, and positive functions) are used to assess the health and welfare of farm animals. Furthermore, we provided an implication of the FMT to improve productivity, health, and welfare of animals. Most importantly, there exist new insights into the microbiota-gut-brain path in order to systemically evaluate and improve animal welfare.

## References

[1] Fraser D., Weary D.M., Pajor E.A., Milligan B.N. (1997). A scientific concept of animal welfare that reflects ethical concerns. Anim. Welf..

[2] Mormede P., Andanson S., Aupérin B., Beerda B., Guémené D., Malmkvist J., Manteca X., Manteuffel G., Prunet P., Veissier I. (2007). Exploration of the hypothalamic-pituitary-adrenal function as a tool to evaluate animal welfare. Physiol. Behav..

[3] Dawkins M.S. (2003). Behaviour as a tool in the assessment of animal welfare. Zoology.

[4] Rushen J., Depassille A.M.B. (1992). The Scientific Assessment of the Impact of Housing on Animal-Welfare—A Critical-Review. Can. J. Anim. Sci..

[5] Botreau R., Bonde M., Butterworth A., Perny P., Bracke M.B.M., Capdeville J., Veissier I. (2007). Aggregation of measures to produce an overall assessment of animal welfare. Part 1: A review of existing methods. Animal.

[6] Akinoso S., Alabi O. Mathematics as an important tool in animal welfare assessments. Proceedings of the International Conference on Animal Welfare.

[7] O’Hara A.M., Shanahan F. (2006). The gut flora as a forgotten organ. EMBO Rep..

[8] Savage D.C. (1977). Microbial ecology of the gastrointestinal tract. Annu. Rev. Microbiol..

[9] Qin J.J., Li R.Q., Raes J.J., Arumugam M., Burgdorf K.S., Manichanh C., Borruel N. (2010). A human gut microbial gene catalogue established by metagenomic sequencing. Nature.

[10] Gill S.R., Pop M., Deboy R.T., Eckburg P.B., Turnbaugh P.J., Samuel B.S., Gordon J.I., Relman D.A., Fraser-Liggett C.M., Nelson K.E. (2006). Metagenomic analysis of the human distal gut microbiome. Science.

[11] Leser T.D., Amenuvor J.Z., Jensen T.K., Lindecrona R.H., Boye M., Moller K. (2002). Culture-independent analysis of gut bacteria: The pig gastrointestinal tract microbiota revisited. Appl. Environ. Microbiol..

[12] Kong Y., Teather R., Forster R. (2010). Composition, spatial distribution, and diversity of the bacterial communities in the rumen of cows fed different forages. FEMS Microbiol. Ecol..

[13] Wei S., Morrison M., Yu Z. (2013). Bacterial census of poultry intestinal microbiome. Poult. Sci..

[14] Apajalahti J., Kettunen A., Graham H. (2004). Characteristics of the gastrointestinal microbial communities, with special reference to the chicken. Worlds Poult. Sci. J..

[15] Ma Y., Ma S., Chang L., Wang H., Ga Q., Ma L., Bai Z., Shen Y., Ge R.L. (2019). Gut microbiota adaptation to high altitude in indigenous animals. Biochem. Biophys. Res. Commun..

[16] Yeoman C.J., White B.A. (2015). Gastrointestinal tract microbiota and probiotics in production animals. Annu. Rev. Anim. Biosci..

[17] Relman D.A. (2012). The human microbiome: Ecosystem resilience and health. Nutr. Rev..

[18] Chen S., Wang J., Peng D., Li G., Chen J., Gu X. (2018). Exposure to heat-stress environment affects the physiology, circulation levels of cytokines, and microbiome in dairy cows. Sci. Rep..

[19] Sergeant M.J., Constantinidou C., Cogan T.A., Bedford M.R., Penn C.W., Pallen M.J. (2014). Extensive microbial and functional diversity within the chicken cecal microbiome. PLoS ONE.

[20] Jiangrang L., Umelaalim I., Barry H., Charles H., Maurer J.J., Lee M.D. (2003). Diversity and succession of the intestinal bacterial community of the maturing broiler chicken. Appl. Environ. Microbiol..

[21] Corrigan A., de Leeuw M., Penaud-Frezet S., Dimova D., Murphy R.A. (2015). Phylogenetic and functional alterations in bacterial community compositions in broiler ceca as a result of mannan oligosaccharide supplementation. Appl. Environ. Microbiol..

[22] Chen S., Xiang H., Zhang H., Zhu X., Wang D., Wang J., Yin T., Liu L., Kong M., Li H. (2019). Rearing system causes changes of behavior, microbiome, and gene expression of chickens. Poult. Sci..

[23] Cryan J.F., Dinan T.G. (2012). Mind-altering microorganisms: The impact of the gut microbiota on brain and behaviour. Nat. Rev. Neurosci..

[24] Bailey M.T., Dowd S.E., Galley J.D., Hufnagle A.R., Allen R.G., Lyte M. (2011). Exposure to a social stressor alters the structure of the intestinal microbiota: Implications for stressor-induced immunomodulation. Brain Behav. Immun..

[25] Neufeld K.M., Kang N., Bienenstock J., Foster J.A. (2011). Reduced anxiety-like behavior and central neurochemical change in germ-free mice. Neurogastroenterol. Motil..

[26] Gareau M.G., Wine E., Rodrigues D.M., Cho J.H., Whary M.T., Philpott D.J., Macqueen G., Sherman P.M. (2011). Bacterial infection causes stress-induced memory dysfunction in mice. Gut.

[27] Kraimi N., Dawkins M., Gebhardt-Henrich S.G., Velge P., Rychlik I., Volf J., Creach P., Smith A., Colles F., Leterrier C. (2019). Influence of the microbiota-gut-brain axis on behavior and welfare in farm animals: A review. Physiol. Behav..

[28] Ridaura V.K., Faith J.J., Rey F.E., Cheng J., Duncan A.E., Kau A.L., Griffin N.W., Lombard V., Henrissat B., Bain J.R. (2013). Gut microbiota from twins discordant for obesity modulate metabolism in mice. Science.

[29] Turnbaugh P.J., Gordon J.I. (2009). The core gut microbiome, energy balance and obesity. J. Physiol..

[30] Backhed F., Ding H., Wang T., Hooper L.V., Koh G.Y., Nagy A., Semenkovich C.F., Gordon J.I. (2004). The gut microbiota as an environmental factor that regulates fat storage. Proc. Natl. Acad. Sci. USA.

[31] Wayman C. (2016). Microbes and the gut–brain axis. Lancet Gastroenterol. Hepatol..

[32] Bercik P., Collins S.M., Verdu E.F. (2012). Microbes and the gut-brain axis. Neurogastroenterol. Motil..

[33] Choct M. (2009). Managing gut health through nutrition. Br. Poult. Sci..

[34] Oakley B.B., Lillehoj H.S., Kogut M.H., Kim W.K., Maurer J.J., Pedroso A., Lee M.D., Collett S.R., Johnson T.J., Cox N.A. (2014). The chicken gastrointestinal microbiome. FEMS Microbiol. Lett..

[35] Gresse R., Chaucheyras-Durand F., Fleury M.A., Van de Wiele T., Forano E., Blanquet-Diot S. (2017). Gut Microbiota Dysbiosis in Postweaning Piglets: Understanding the Keys to Health. Trends Microbiol..

[36] Min B.R., Solaiman S. (2018). Comparative aspects of plant tannins on digestive physiology, nutrition and microbial community changes in sheep and goats: A review. J. Anim. Physiol. Anim. Nutr..

[37] Núria M., Aline F., Sandra K., Fabrice R., Marco M., Maria B., Diane E., Julie R., Guillaume S., Philippe G. (2017). The effects of weaning methods on gut microbiota composition and horse physiology. Front. Physiol..

[38] Kraimi N., Calandreau L., Biesse M., Rabot S., Guitton E., Velge P., Leterrier C. (2018). Absence of Gut Microbiota Reduces Emotional Reactivity in Japanese Quails (*Coturnix japonica*). Front. Physiol..

[39] Palmer M.F., Rolls B.A. (1983). The Activities of Some Metabolic Enzymes in the Intestines of Germ-Free and Conventional Chicks. Br. J. Nutr..

[40] Hu J., Lin S., Zheng B., Cheung P.C.K. (2018). Short-chain fatty acids in control of energy metabolism. Crit. Rev. Food Sci. Nutr..

[41] Herath M., Hosie S., Bornstein J.C., Franks A.E., Hill-Yardin E.L. (2020). The role of the gastrointestinal mucus system in intestinal homeostasis: Implications for neurological disorders. Front. Cell. Infect. Microbiol..

[42] Zhou X., Jiang X., Yang C., Ma B., Lei C., Xu C., Zhang A., Yang X., Xiong Q., Zhang P. (2016). Cecal microbiota of Tibetan Chickens from five geographic regions were determined by 16S rRNA sequencing. Microbiologyopen.

[43] Chen S., Xiang H., Zhu X., Zhang H., Wang D., Liu H., Wang J., Yin T., Liu L., Kong M. (2018). Free Dietary Choice and Free-Range Rearing Improve the Product Quality, Gait Score, and Microbial Richness of Chickens. Animals.

[44] Yan C., Xiao J., Chen D., Turner S.P., Zhao X. (2021). Feed Restriction Induced Changes in Behavior, Corticosterone, and Microbial Programming in Slow- and Fast-Growing Chicken Breeds. Animals.

[45] Wang X., Tsai T., Deng F., Wei X., Chai J., Knapp J., Apple J., Maxwell C.V., Lee J.A., Li Y. (2019). Longitudinal investigation of the swine gut microbiome from birth to market reveals stage and growth performance associated bacteria. Microbiome.

[46] Gomez D.E., Arroyo L.G., Costa M.C., Viel L., Weese J.S. (2017). Characterization of the Fecal Bacterial Microbiota of Healthy and Diarrheic Dairy Calves. J. Vet. Intern. Med..

[47] Pandit R.J., Hinsu A.T., Patel N.V., Koringa P.G., Jakhesara S.J., Thakkar J.R., Shah T.M., Limon G., Psifidi A., Guitian J. (2018). Microbial diversity and community composition of caecal microbiota in commercial and indigenous Indian chickens determined using 16s rDNA amplicon sequencing. Microbiome.

[48] Furness J.B., Kunze W.A.A., Clerc N. (1999). Nutrient tasting and signaling mechanisms in the gut II. The intestine as a sensory organ: Neural, endocrine, and immune responses. Am. J. Physiol..

[49] Round J.L., O’Connell R.M., Mazmanian S.K. (2010). Coordination of tolerogenic immune responses by the commensal microbiota. J. Autoimmun..

[50] Torsten O., Dingding A., Sebastian Z., Miguel Pinilla V., Julia R., Andre F., Glickman J.N., Reiner S., Baron R.M., Kasper D.L. (2012). Microbial exposure during early life has persistent effects on natural killer T cell function. Inflamm. Bowel Dis. Monit..

[51] Martin R., Nauta A.J., Ben Amor K., Knippels L.M., Knol J., Garssen J. (2010). Early life: Gut microbiota and immune development in infancy. Benef. Microbes.

[52] O’Mahony S.M., Marchesi J.R., Scully P., Codling C., Ceolho A.M., Quigley E.M., Cryan J.F., Dinan T.G. (2009). Early life stress alters behavior, immunity, and microbiota in rats: Implications for irritable bowel syndrome and psychiatric illnesses. Biol. Psychiatry.

[53] Kayama H., Takeda K. (2016). Functions of innate immune cells and commensal bacteria in gut homeostasis. J. Biochem..

[54] Troy E.B., Kasper D.L. (2010). Beneficial effects of Bacteroides fragilis polysaccharides on the immune system. Front. Biosci..

[55] Bauer E., Williams B.A., Smidt H., Verstegen M.W., Mosenthin R. (2006). Influence of the gastrointestinal microbiota on development of the immune system in young animals. Curr. Issues Intest. Microbiol..

[56] Hegde S.N., Rolls B.A., Turvey A., Coates M.E. (1982). Influence of Gut Microflora on the Lymphoid-Tissue of the Chicken (*Gallus-domesticus*) and Japanese Quail (*Coturnix-coturnix-japonica*). Comp. Biochem. Physiol. Part A Physiol..

[57] Cheled-Shoval S.L., Gamage N.S., Amit-Romach E., Forder R., Marshal J., Van Kessel A., Uni Z. (2014). Differences in intestinal mucin dynamics between germ-free and conventionally reared chickens after mannan-oligosaccharide supplementation. Poult. Sci..

[58] Richards P.J., Flaujac Lafontaine G.M., Connerton P.L., Liang L., Asiani K., Fish N.M., Connerton I.F. (2020). Galacto-Oligosaccharides Modulate the Juvenile Gut Microbiome and Innate Immunity To Improve Broiler Chicken Performance. mSystems.

[59] Zenner C., Hitch T.C.A., Riedel T., Wortmann E., Tiede S., Buhl E.M., Abt B., Neuhaus K., Velge P., Overmann J. (2021). Early-Life Immune System Maturation in Chickens Using a Synthetic Community of Cultured Gut Bacteria. mSystems.

[60] Jin Y.B., Cao X., Shi C.W., Feng B., Huang H.B., Jiang Y.L., Wang J.Z., Yang G.L., Yang W.T., Wang C.F. (2021). Lactobacillus rhamnosus GG Promotes Early B Lineage Development and IgA Production in the Lamina Propria in Piglets. J. Immunol..

[61] Trebichavsky I., Schulze J., Dlabac V., Cukrowska B., Tlaskalova-Hogenova H., Rehakova Z. (1998). Salmonellosis: Lessons drawn from a germ-free pig model. Folia Microbiol..

[62] Haverson K., Rehakova Z., Sinkora J., Sver L., Bailey M. (2007). Immune development in jejunal mucosa after colonization with selected commensal gut bacteria: A study in germ-free pigs. Vet. Immunol. Immunopathol..

[63] Malmuthuge N., Li M., Goonewardene L.A., Oba M., Guan L.L. (2013). Effect of calf starter feeding on gut microbial diversity and expression of genes involved in host immune responses and tight junctions in dairy calves during weaning transition. J. Dairy Sci..

[64] Chung H., Pamp S.J., Hill J.A., Surana N.K., Edelman S.M., Troy E.B., Reading N.C., Villablanca E.J., Wang S., Mora J.R. (2012). Gut immune maturation depends on colonization with a host-specific microbiota. Cell.

[65] Lima-Ojeda J.M., Rupprecht R., Baghai T.C. (2020). Gut microbiota and depression: Pathophysiology of depression: Hypothalamic-pituitary-adrenal axis and microbiota-gut-brain axis. Nervenarzt.

[66] Vuong H.E., Yano J.M., Fung T.C., Hsiao E.Y. (2017). The Microbiome and Host Behavior. Annu. Rev. Neurosci..

[67] Azeem N.A. (2013). Do probiotics affect the behavior of turkey poults?. J. Vet. Med. Anim. Health.

[68] Goodfellow C.K., Whitney T., Christie D.M., Sicotte P., Wikberg E.C., Ting N. (2019). Divergence in gut microbial communities mirrors a social group fission event in a black-and-white colobus monkey (*Colobus vellerosus*). Am. J. Primatol..

[69] Nunn C.L., Altizer S.M. (2006). Infectious Diseases in Primates: Behavior, Ecology and Evolution.

[70] Altizer S., Nunn C.L., Thrall P.H., Gittleman J.L., Antonovics J., Cunningham A.A., Dobson A.P., Ezenwa V., Jones K.E., Pedersen A.B. (2003). Social organization and parasite risk in mammals: Integrating theory and empirical studies. Annu. Rev. Ecol. Evol. Syst..

[71] Loehle C. (1995). Social Barriers to Pathogen Transmission in Wild Animal Populations. Ecology.

[72] Banning J.L., Weddle A.L., Wahl G.W., Simon M.A., Lauer A., Walters R.L., Harris R.N. (2008). Antifungal skin bacteria, embryonic survival, and communal nesting in four-toed salamanders, Hemidactylium scutatum. Oecologia.

[73] Gunderson A.R., Forsyth M.H., Swaddle J.P. (2010). Evidence that plumage bacteria influence feather coloration and body condition of eastern bluebirds *Sialia sialis*. J. Avian Biol..

[74] Toscano M.J., Sait L., Jorgensen F., Nicol C.J., Powers C., Smith A.L., Bailey M., Humphrey T.J. (2010). Sub-clinical infection with Salmonella in chickens differentially affects behaviour and welfare in three inbred strains. Br. Poult. Sci..

[75] Meyer B., Zentek J., Harlander-Matauschek A. (2013). Differences in intestinal microbial metabolites in laying hens with high and low levels of repetitive feather-pecking behavior. Physiol. Behav..

[76] Van der Eijk J.A.J., de Vries H., Kjaer J.B., Naguib M., Kemp B., Smidt H., Rodenburg T.B., Lammers A. (2019). Differences in gut microbiota composition of laying hen lines divergently selected on feather pecking. Poult. Sci..

[77] Birkl P., Bharwani A., Kjaer J.B., Kunze W., McBride P., Forsythe P., Harlander-Matauschek A. (2018). Differences in cecal microbiome of selected high and low feather-pecking laying hens. Poult. Sci..

[78] Leclaire S., Czirjak G.A., Hammouda A., Gasparini J. (2015). Feather bacterial load shapes the trade-off between preening and immunity in pigeons. BMC Evol. Biol..

[79] Yang H., Yang M., Fang S., Huang X., He M., Ke S., Gao J., Wu J., Zhou Y., Fu H. (2018). Evaluating the profound effect of gut microbiome on host appetite in pigs. BMC Microbiol..

[80] Val-Laillet D. (2019). Review: Impact of food, gut-brain signals and metabolic status on brain activity in the pig model: 10 years of nutrition research using in vivo brain imaging. Animal.

[81] Val-Laillet D., Besson M., Guerin S., Coquery N., Randuineau G., Kanzari A., Quesnel H., Bonhomme N., Bolhuis J.E., Kemp B. (2017). A maternal Western diet during gestation and lactation modifies offspring’s microbiota activity, blood lipid levels, cognitive responses, and hippocampal neurogenesis in Yucatan pigs. FASEB J..

[82] Kohari D., Sato S., Nakai Y. (2009). Does the maternal grooming of cattle clean bacteria from the coat of calves?. Behav. Processes.

[83] Whiteside S.A., Razvi H., Dave S., Reid G., Burton J.P. (2015). The microbiome of the urinary tract--a role beyond infection. Nat. Rev. Urol..

[84] Molina-Torres G., Rodriguez-Arrastia M., Roman P., Sanchez-Labraca N., Cardona D. (2019). Stress and the gut microbiota-brain axis. Behav. Pharmacol..

[85] Wiley N.C., Dinan T.G., Ross R.P., Stanton C., Clarke G., Cryan J.F. (2017). The microbiota-gut-brain axis as a key regulator of neural function and the stress response: Implications for human and animal health. J. Anim. Sci..

[86] Tannock G.W., Savage D.C. (1974). Influences of dietary and environmental stress on microbial populations in the murine gastrointestinal tract. Infect. Immun..

[87] Sudo N., Chida Y., Aiba Y., Sonoda J., Oyama N., Yu X.N., Kubo C., Koga Y. (2004). Postnatal microbial colonization programs the hypothalamic-pituitary-adrenal system for stress response in mice. J. Physiol..

[88] Vodičkaa M., Erganga P., Hrnčířb T., Mikuleckáa A., Kvapilováa P. (2018). Microbiota affects the expression of genes involved in HPA axis regulation and local metabolism of glucocorticoids in chronic psychosocial stress. Brain. Behav. Immun..

[89] Ran H., Zeng B., Li Z., Ke C., Bo L., Luo Y., Wang H., Zhou C., Liang F., Li W. (2017). Microbiota Modulate Anxiety-Like Behavior and Endocrine Abnormalities in Hypothalamic-Pituitary-Adrenal Axis. Front. Cell. Infect. Microbiol..

[90] Dan J., Yaalon D.H., Koyumdjisky H. (2002). Environmental enrichment reverses the effects of maternal separation on stress reactivity. J. Neurosci..

[91] O’Mahony S.M., Hyland N.P., Dinan T.G., Cryan J.F. (2011). Maternal separation as a model of brain-gut axis dysfunction. Psychopharmacology.

[92] Bailey M.T., Coe C.L. (2015). Maternal separation disrupts the integrity of the intestinal microflora in infant rhesus monkeys. Dev. Psychobiol..

[93] Vlčková K., Shutt-Phillips K., Heistermann M., Pafčo B., Gomez A. (2017). Impact of stress on the gut microbiome of free-ranging western lowland gorillas. Microbiology.

[94] Burkholder K.M., Thompson K.L., Einstein M.E., Applegate T.J., Patterson J.A. (2008). Influence of stressors on normal intestinal microbiota, intestinal morphology, and susceptibility to Salmonella enteritidis colonization in broilers. Poult. Sci..

[95] Yan C., Hartcher K., Liu W., Xiao J., Xiang H., Wang J., Liu H., Zhang H., Liu J., Chen S. (2020). Adaptive response to a future life challenge: Consequences of early-life environmental complexity in dual-purpose chicks. J. Anim. Sci..

[96] Wu Q., Xu Z.Y., Song S.Y., Zhang H., Zhang W.Y., Liu L.P., Chen Y.P., Sun J.H. (2020). Gut microbiota modulates stress-induced hypertension through the HPA axis. Brain Res. Bull..

[97] O’Callaghan T.F., Ross R.P., Stanton C., Clarke G. (2016). The gut microbiome as a virtual endocrine organ with implications for farm and domestic animal endocrinology. Domest. Anim. Endocrinol..

[98] Khoruts A., Staley C., Sadowsky M.J. (2020). Faecal microbiota transplantation for Clostridioides difficile: Mechanisms and pharmacology. Nat. Rev. Gastroenterol. Hepatol..

[99] Khoruts A., Sadowsky M.J. (2016). Understanding the mechanisms of faecal microbiota transplantation. Nat. Rev. Gastroenterol. Hepatol..

[100] Shankar V., Hamilton M.J., Khoruts A., Kilburn A., Unno T., Paliy O., Sadowsky M.J. (2014). Species and genus level resolution analysis of gut microbiota in Clostridium difficile patients following fecal microbiota transplantation. Microbiome.

[101] Seekatz A.M., Aas J., Gessert C.E., Rubin T.A., Saman D.M., Bakken J.S., Young V.B. (2014). Recovery of the gut microbiome following fecal microbiota transplantation. mBio.

[102] Weingarden A., Gonzalez A., Vazquez-Baeza Y., Weiss S., Humphry G., Berg-Lyons D., Knights D., Unno T., Bobr A., Kang J. (2015). Dynamic changes in short- and long-term bacterial composition following fecal microbiota transplantation for recurrent Clostridium difficile infection. Microbiome.

[103] Borody T.J., Khoruts A. (2012). Fecal microbiota transplantation and emerging applications. Nat. Rev. Gastroenterol. Hepatol..

[104] Faming Z., Wensheng L., Yan S., Zhining F., Guozhong J. (2012). Should we standardize the 1,700-year-old fecal microbiota transplantation?. Am. J. Gastroenterol..

[105] Zhang F., Cui B., He X., Nie Y., Wu K., Fan D., Group F.M.-s.S. (2018). Microbiota transplantation: Concept, methodology and strategy for its modernization. Protein Cell.

[106] Surawicz C.M., Brandt L.J., Binion D.G., Ananthakrishnan A.N., Curry S.R., Gilligan P.H., McFarland L.V., Mellow M., Zuckerbraun B.S. (2013). Guidelines for diagnosis, treatment, and prevention of Clostridium difficile infections. Am. J. Gastroenterol..

[107] Klingensmith N.J., Coopersmith C.M. (2016). Fecal microbiota transplantation for multiple organ dysfunction syndrome. Crit. Care.

[108] Moayyedi P., Surette M.G., Kim P.T., Libertucci J., Wolfe M., Onischi C., Armstrong D., Marshall J.K., Kassam Z., Reinisch W. (2015). Fecal Microbiota Transplantation Induces Remission in Patients with Active Ulcerative Colitis in a Randomized Controlled Trial. Gastroenterology.

[109] Rossen N.G., Fuentes S., van der Spek M.J., Tijssen J.G., Hartman J.H., Duflou A., Lowenberg M., van den Brink G.R., Mathus-Vliegen E.M., de Vos W.M. (2015). Findings from a Randomized Controlled Trial of Fecal Transplantation for Patients with Ulcerative Colitis. Gastroenterology.

[110] Kang D.W., Adams J.B., Gregory A.C., Borody T., Chittick L., Fasano A., Khoruts A., Geis E., Maldonado J., McDonough-Means S. (2017). Microbiota Transfer Therapy alters gut ecosystem and improves gastrointestinal and autism symptoms: An open-label study. Microbiome.

[111] Naigles L.R., Johnson R., Mastergeorge A., Ozonoff S., Rogers S.J., Amaral D.G., Nordahl C.W. (2017). Neural correlates of language variability in preschool-aged boys with autism spectrum disorder. Autism Res..

[112] Schwartz M., Gluck M., Koon S. (2013). Norovirus gastroenteritis after fecal microbiota transplantation for treatment of Clostridium difficile infection despite asymptomatic donors and lack of sick contacts. Am. J. Gastroenterol..

[113] Abautret-Daly A., Dempsey E., Parra-Blanco A., Medina C., Harkin A. (2018). Gut-brain actions underlying comorbid anxiety and depression associated with inflammatory bowel disease. Acta Neuropsychiatr..

[114] Zheng P., Zeng B., Zhou C., Liu M., Fang Z., Xu X., Zeng L., Chen J., Fan S., Du X. (2016). Gut microbiome remodeling induces depressive-like behaviors through a pathway mediated by the host’s metabolism. Mol. Psychiatry.

[115] Kulecka M., Paziewska A., Zeber-Lubecka N., Ambrozkiewicz F., Kopczynski M., Kuklinska U., Pysniak K., Gajewska M., Mikula M., Ostrowski J. (2016). Prolonged transfer of feces from the lean mice modulates gut microbiota in obese mice. Nutr. Metab..

[116] De Palma G., Lynch M.D., Lu J., Dang V.T., Deng Y., Jury J., Umeh G., Miranda P.M., Pigrau Pastor M., Sidani S. (2017). Transplantation of fecal microbiota from patients with irritable bowel syndrome alters gut function and behavior in recipient mice. Sci. Transl. Med..

[117] Odle J., Lin X., Jacobi S.K., Kim S.W., Stahl C.H. (2014). The Suckling Piglet as an Agrimedical Model for the Study of Pediatric Nutrition and Metabolism. Annu. Rev. Anim. Biosci..

[118] Guilloteau P., Zabielski R., Hammon H.M., Metges C.C. (2010). Nutritional programming of gastrointestinal tract development. Is the pig a good model for man?. Nutr. Res. Rev..

[119] Weimer P.J., Stevenson D.M., Mantovani H.C., Man S.L. (2010). Host specificity of the ruminal bacterial community in the dairy cow following near-total exchange of ruminal contents. J. Dairy Sci..

[120] Ji S., Jiang T., Yan H., Guo C., Liu J., Su H., Alugongo G.M., Shi H., Wang Y., Cao Z. (2018). Ecological Restoration of Antibiotic-Disturbed Gastrointestinal Microbiota in Foregut and Hindgut of Cows. Front. Cell Infect. Microbiol..

[121] Ma C., Sun Z., Zeng B., Huang S., Zhao J., Zhang Y., Su X., Xu J., Wei H., Zhang H. (2018). Cow-to-mouse fecal transplantations suggest intestinal microbiome as one cause of mastitis. Microbiome.

[122] Hu J., Ma L., Nie Y., Chen J., Zheng W., Wang X., Xie C., Zheng Z., Wang Z., Yang T. (2018). A Microbiota-Derived Bacteriocin Targets the Host to Confer Diarrhea Resistance in Early-Weaned Piglets. Cell Host Microbe.

[123] Geng S., Cheng S., Li Y., Wen Z., Ma X., Jiang X., Wang Y., Han X. (2018). Faecal Microbiota Transplantation Reduces Susceptibility to Epithelial Injury and Modulates Tryptophan Metabolism of the Microbial Community in a Piglet Model. J. Crohns Colitis.

[124] Yan H.L., Diao H., Xiao Y., Li W.X., Yu B., He J., Yu J., Zheng P., Mao X.B., Luo Y.H. (2016). Gut microbiota can transfer fiber characteristics and lipid metabolic profiles of skeletal muscle from pigs to germ-free mice. Sci. Rep..

[125] Cheng S., Ma X., Geng S., Jiang X., Li Y., Hu L., Li J., Wang Y., Han X. (2018). Fecal microbiota transplantation beneficially regulates intestinal mucosal autophagy and alleviates gut barrier injury. mSystems.

[126] Hu L., Geng S., Li Y., Cheng S., Fu X., Yue X., Han X. (2017). Exogenous Fecal Microbiota Transplantation from Local Adult Pigs to Crossbred Newborn Piglets. Front. Microbiol..

[127] Nurmi E., Rantala M. (1973). New aspects of Salmonella infection in broiler production. Nature.

[128] Donaldson E.E., Stanley D., Hughes R.J., Moore R.J. (2017). The time-course of broiler intestinal microbiota development after administration of cecal contents to incubating eggs. PeerJ.

[129] Siegerstetter S.C., Petri R.M., Magowan E., Lawlor P.G., Zebeli Q., O’Connell N.E., Metzler-Zebeli B.U. (2018). Fecal Microbiota Transplant from Highly Feed-Efficient Donors Shows Little Effect on Age-Related Changes in Feed-Efficiency-Associated Fecal Microbiota from Chickens. Appl. Environ. Microbiol..

[130] Yan C., Xiao J., Li Z., Liu H., Zhao X., Liu J., Chen S., Zhao X. (2021). Exogenous Fecal Microbial Transplantation Alters Fearfulness, Intestinal Morphology, and Gut Microbiota in Broilers. Front. Vet. Sci..

[131] Kraimi N., Calandreau L., Zemb O., Germain K., Dupont C., Velge P., Guitton E., Lavillatte S., Parias C., Leterrier C. (2019). Effects of gut microbiota transfer on emotional reactivity in Japanese quails (*Coturnix japonica*). J. Exp. Biol..

[132] Choi H.H., Cho Y.S. (2016). Fecal Microbiota Transplantation: Current Applications, Effectiveness, and Future Perspectives. Clin. Endosc..

[133] Stollman N., Smith M., Giovanelli A., Mendolia G., Burns L., Didyk E., Burgess J., Noh A., Edelstein C., Alm E. (2015). Frozen encapsulated stool in recurrent Clostridium difficile: Exploring the role of pills in the treatment hierarchy of fecal microbiota transplant nonresponders. Am. J. Gastroenterol..

[134] Rubin T.A., Gessert C.E., Aas J., Bakken J.S. (2013). Fecal microbiome transplantation for recurrent Clostridium difficile infection: Report on a case series. Anaerobe.

[135] Mattila E., Uusitalo-Seppala R., Wuorela M., Lehtola L., Nurmi H., Ristikankare M., Moilanen V., Salminen K., Seppala M., Mattila P.S. (2012). Fecal transplantation, through colonoscopy, is effective therapy for recurrent Clostridium difficile infection. Gastroenterology.

[136] Diao H., Yan H.L., Xiao Y., Yu B., Yu J., He J., Zheng P., Zeng B.H., Wei H., Mao X.B. (2016). Intestinal microbiota could transfer host Gut characteristics from pigs to mice. BMC Microbiol..

[137] Zhou M., Peng Y.J., Chen Y., Klinger C.M., Oba M., Liu J.X., Guan L.L. (2018). Assessment of microbiome changes after rumen transfaunation: Implications on improving feed efficiency in beef cattle. Microbiome.

[138] DePeters E.J., George L.W. (2014). Rumen transfaunation. Immunol. Lett..

